# Cerebrospinal Fluid Total and Phosphorylated Tau Protein in Behavioral Variant Frontotemporal Dementia, Progressive Supranuclear Palsy, Corticobasal Syndrome and Non-Fluent Agrammatic Primary Progressive Aphasia: A Systematic Review and Meta-Analysis

**DOI:** 10.3390/biomedicines12081781

**Published:** 2024-08-06

**Authors:** Nikolaos Giagkou, Ioanna Kapsali, Maria-Evgenia Brinia, Vasilios C. Constantinides

**Affiliations:** 1 Neurodegenerative Disorders and Epilepsy Ward, First Department of Neurology, National and Kapodistrian University of Athens, Eginition Hospital, 11528 Athens, Greeceioannakapsali@ymail.com (I.K.); mariaevgeniabr@gmail.com (M.-E.B.); 2 Neurochemistry and Biomarkers Unit, First Department of Neurology, National and Kapodistrian University of Athens, Eginition Hospital, 11528 Athens, Greece

**Keywords:** tau protein, phosphorylated tau protein, cerebrospinal fluid, biomarkers, frontotemporal dementia, progressive supranuclear palsy, corticobasal syndrome, primary progressive aphasia, tauopathies

## Abstract

(1) Background: Frontotemporal lobar degeneration (FTLD) is a generic term which refers to multiple pathologies, including FTLD-tau. The most common FTLD-tau diseases are Pick’s disease (PiD), progressive supranuclear palsy (PSP) and corticobasal degeneration (CBD). These diseases share four major syndromes: behavioral variant frontotemporal dementia (bvFD), Richardson syndrome (RS), corticobasal syndrome (CBS) and non-fluent agrammatic primary progressive aphasia (nfa-PPA). The primary aim of this meta-analysis was to examine the diagnostic performance of CSF total (t-tau) and phosphorylated (p-tau) protein in bvFTD, RS, CBS, nfa-PPA and pathologically or genetically defined tauopathy. (2) Methods: A systematic review and meta-analysis was performed on all studies with >10 subjects in a bvFTD/RS/CBS/nfa-PPA group and control group and available data on CSF t-tau or p-tau (mean, SD). Cohen’s *d* was used to quantify the effect size of each study (3) Results: The PSP/tauopathy patients exhibited decreased levels of CSF p-tau compared to the control subjects. The CBS/bvFTD/nfa-PPA cohorts exhibited an increase in t-tau compared to the control groups. (4) Conclusions: Tauopathies may exhibit an inherent decrease in CSF p-tau. The admixture of AD patients in FTD cohorts and high heterogeneity among studies on rare diseases are significant confounding factors in FTLD studies.

## 1. Introduction

Frontotemporal dementia (FTD) is a generic term used to describe a variety of diverse clinical phenotypes which are caused by frontotemporal lobar degeneration (FTLD). FTLD can be subdivided into four major groups, depending on the underlying proteinopathy, which include FTLD-tau, FTLD-TDP (TAR DNA-binding protein 43), FTLD-FUS (fused in sarcoma) and FTLD-UPS (ubiquitin proteasome system) [1]. Within the spectrum of FTL-tau, the most common neuropathological entities are Pick’s disease (PiD), a 3R-tauopathy; progressive supranuclear palsy (PSP); and corticobasal degeneration (CBD), (both of which are 4R-tauopathies) [2].

PiD most commonly manifests with behavioral variant FTD (bvFTD) or non-fluent agrammatic primary progressive aphasia (nfa-PPA) [3,4]. PSP exhibits a wide phenotypical variability, manifesting with Richardson syndrome, primary gait freezing (PSP-PGF), PSP with predominant parkinsonism (PSP-P), as well as with predominantly cognitive phenotypes (frontal presentation: PSP-F; predominant speech/language disorder: PSP-SL; corticobasal syndrome: PSP-CBS) [5]. Likewise, CBD manifests with CBS, a frontal behavioral–spatial syndrome, as nfa-PPA and as a PSP syndrome [6]. Despite this clinical heterogeneity, it seems that the three major FTLD-tauopathies converge into four overlapping phenotypes: bvFTD, RS, CBS and nfa-PPA.

In accordance with the great clinical variability of distinct neuropathological entities, there is also great neuropathological variability when confronted with each of the classical FTD phenotypes. Thus, a bvFTD phenotype may be due to an underlying FTLD-tau pathology (~40–50%), FTLD-TDP pathology (40–50%), Alzheimer’s disease (AD) or other rarer pathologies [7]. Likewise, CBS most commonly has a 4R-tauopathy (CBD or PSP) as its underlying pathology. However, ~25–40% of CBS patients have an underlying AD pathology [8]. Patients with nfa-PPA most commonly harbor an FTLD-tau pathology, followed by FTLD-TDP and rarely AD [9]. Contrary to bvFTD, CBS and nfa-PPA, patients with RS almost always harbor a 4R-tauopathy [10].

Given this great clinical–pathological overlap among FTD phenotypes, a biomarker with molecular specificity for an underlying tau pathology is paramount for the correct in vivo diagnosis of tauopathies. This would greatly improve the accuracy of patient stratification in clinical trials of candidate disease-modifying agents, and enhance the clinical management of FTD patients.

To this end, several studies have assessed the diagnostic accuracy of total and phosphorylated tau (t-tau and p-tau, respectively) proteins in the cerebrospinal fluid (CSF) as candidate markers of tau pathology. Multiple lines of evidence from the field of AD have established that t-tau is a non-specific marker of neurodegeneration, whereas p-tau is a specific marker of tau pathology in patients with AD [11]. However, variations of t-tau and p-tau have been documented in diverse neurodegenerative disorders, including FTD [12,13,14].

To date, a single meta-analysis has been performed with regard to t-tau and p-tau in FTLD [15]. This meta-analysis was performed over a decade ago, and aimed at comparing these biomarkers between FTLD and Alzheimer’s disease. The aim of the present systematic review and meta-analysis is to present the existing data on CSF t-tau and p-tau in cohorts of bvFTD, PSP, CBS and nfa-PPA patients, and to examine the diagnostic performance of these markers in identifying tau pathology.

## 2. Materials and Methods

The PRISMA statement (Preferred Reporting Items for Systematic Reviews and Meta-Analyses) was followed for this meta-analysis (Supplementary File S1) [16]. The study protocol was registered in PROSPERO (ID: CRD42024557016). No institutional board review approval was obtained, since this study did not utilize novel data and was based exclusively on previously published data.

### 2.1. Literature Search Strategy

PubMed, Scopus and Web of Science Core Collection were searched from database inception to 1 April 2024 by three researchers independently (N.G., I.K. and M.-E.B.). A consensus among reviewers regarding study eligibility was required for study inclusion. After this initial search, a search was performed manually on all included studies, which included (a) citations of included studies; (b) references of included studies; (c) most relevant studies (from PubMed); (d) studies included in systematic reviews and meta-analyses performed in the past. In cases of full-text unavailability, corresponding authors of papers were contacted.

The search strategy applied was as follows:

( (corticobasal) OR (aphasia) OR (supranuclear) OR (Richardson) OR (behavioral) OR (behavioural) OR (frontotemporal) OR (agrammatic) OR (tauopath*)) AND ( (biomarker*) OR (p-tau) OR (ptau) OR (tau) OR (phosphorylated tau)) AND ( (cerebrospinal fluid) OR (fluid) OR (csf)

in the study title or abstract.

The search strategy is summarized in Supplementary File S2.

### 2.2. Eligibility Criteria and Study Selection

Inclusion criteria were as follows:

(a) Publication in English language;
(b) Original, peer-reviewed research papers;
(c) Studies with ≥10 subjects in at least one of the patient groups (behavioral variant frontotemporal dementia (bvFTD); progressive supranuclear pasly (PSP); corticobasal syndrome (CBS); non-fluent agrammatic variant of primary progressive aphasia (nfa-PPA)) and ≥10 control subjects. Regarding bvFTD, all patients who fulfilled the Lund and Manchester [17] and the Rascovsky [4] criteria were included. When the Neary criteria were implemented [18], patients were included only in cases where there was a clear distinction between the behavioral variant and progressive aphasias. Regarding tauopathy definition, patients were included when there was neuropathological confirmation of the diagnosis or genetically confirmed cases (pathogenic MAPT mutation).
(d) Studies with available data on at least one of total CSF tau protein (t-tau) or phosphorylated CSF tau protein (p-tau);
(e) Studies with available/retrievable mean values (mean) and standard deviations (SDs) of CSF t-tau or p-tau.

Exclusion criteria were as follows:

(a) Non-original studies (reviews, meta-analyses);
(b) Case series with <10 subjects in patient groups or <10 control subjects;
(c) Case reports;
(d) Abstracts in conferences, congresses of other scientific meetings;
(e) Studies with identical patient cohorts.

In cases of publications with complete or partial overlap of patient cohorts, the most recent study or the study with the largest samples was selected, based on an algorithm regarding duplicate studies [19]. This algorithm includes study characteristics, evaluation of authorship, definition of constructs, sample characteristics and measures and study effects.

### 2.3. Data Extraction

Data extraction was performed by three authors independently (N.G., I.K. and M-E.B.). In cases of data discrepancies among databases, raw data from the manuscripts were re-evaluated.

Information extracted from studies included the following study characteristics: year of study; author; study ID (author, year); study title; patient recruitment period; study site (s); study design (i.e., prospective, retrospective, cross-sectional, longitudinal, not specified); type of control group (i.e., healthy controls vs. neurological controls).

Data extracted regarding the patient groups and control group, where applicable, were as follows: n (number of subjects); male/female ratio; mean age (y); mean disease duration (y); mean CSF t-tau per group; standard deviation (SD) of CSF t-tau per group; mean CSF p-tau per group; SD of CSF p-tau per group.

In cases of missing (not reported) data, a review of the supplementary files of relevant papers was performed. When t-tau or p-tau were presented as medians (interquartile range), data were not extracted from boxplots, scatterplots or error bar plots based on the assumption that these cohorts did not fulfill criteria for normal distribution/equal variances.

### 2.4. Summary Measures

Cohen’s *d*, as a measure of standardized mean difference (SMD), was calculated to quantify the effect size of total and phosphorylated CSF tau protein levels for the distinction of bvFTD, PSP, CBS and nfa-PPA patients from control subjects. Effect size, as measured by Cohen’s *d*, was evaluated as very small (d ≈ 0.01); small (d ≈ 0.2); medium (d ≈ 0.5); large (d ≈ 0.8); very large (d ≈ 1.2); or huge (d ≈ 2.0), based on relevant recommendations [20].

### 2.5. Quality Evaluation

Three authors performed quality evaluation independently (I.K., M-E.B., N.G.), implementing the QUADAS-2 tool [21]. QUADAS-2 includes four key domains: patient selection; index test; reference standard; flow/timing. These domains are evaluated regarding concerns relating to applicability and bias. For the purposes of the present meta-analysis, the following signaling questions were excluded due to non-applicability: (a) “Was a case–control design avoided?” was excluded from the patient selection domain, since this meta-analysis includes, by definition, case–control studies; (b) “If a threshold was used, was it pre-specified?” was removed from the index test domain, since this was not relevant to the design of this meta-analysis; (c) “Was there an appropriate interval between index tests and reference standard?” was removed from the flow and timing domain (non-applicable).

Even when data regarding the signaling questions “Were the index test results interpreted without knowledge of the results of the reference standard” and “Were the reference standard results interpreted without knowledge of the results of the index test” were not specified or missing, this was not considered to significantly affect the applicability or increase the risk of bias of a study, considering that bvFTD, PSP, CBS and nfa-PPA diagnosis is clinical and CSF t-tau and p-tau measurement cannot be manipulated.

### 2.6. Statistical Analysis

Qualitative assessment of the presence/absence of heterogeneity was performed using the *Q* statistic. Between-study heterogeneity was quantified by the *I^2^* statistic. Based on the *I^2^* value, heterogeneity was classified as low (<25%); moderate (25–50%); and high (>50%). For meta-analysis, a random effects model was applied in an effort to control for between-study heterogeneity. As a measure of the effect size of the distinction between CSF t-tau and p-tau levels in bvFTD, PSP, CBD, nfa-PPA patients and control groups, Cohen’s *d* was applied. Forest plots were used to illustrate effect sizes, standard errors, confidence interval limits, *p*-values and weights.

Funnel plots were used to test for publication bias, (x-axis: Cohen’s *d*; y-axis: standard error) in order to visualize any outlying studies. Egger linear regression test was applied in order to quantify bias.

SPSS vs. 29 (IBM Corp. Released 2023; IBM SPSS Statistics for Windows, Version 29.0. Armonk, NY, USA: IBM Corp) was used by one author (V.C.C.) for all statistical analyses. A two-tailed *p* value < 0.05 was defined as the threshold of statistical significance.

## 3. Results

### 3.1. Literature Search and Screening Results

**Figure 1:**
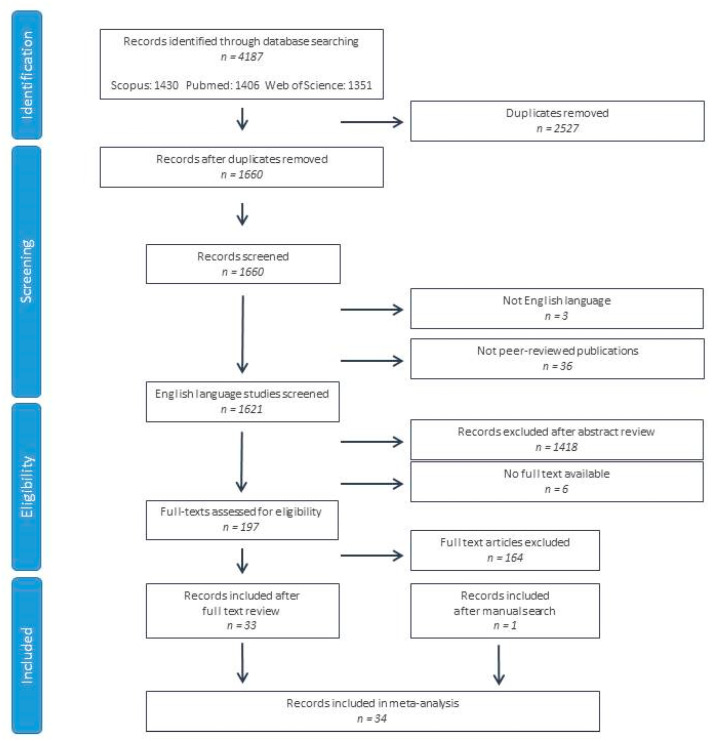
Flow chart of study selection according to PRISMA criteria.

A total of 4187 papers were retrieved from the PubMed, Scopus and Web of Science databases. After the elimination of duplicate records, 1660 records were screened by a review of the title and abstract. Three studies were eliminated due to being written in a non-English language, 36 due to non-peer-reviewed publications, 6 due to the non-availability of the full text, and 1418 after the abstract review. A total of 19 studies were reviewed based on their full texts, resulting in the exclusion of 164 further studies. In total, 34 studies were included in the systematic review and meta-analysis (33 after a full-text review and a single study after a manual search) [22,23,24,25,26,27,28,29,30,31,32,33,34,35,36,37,38,39,40,41,42,43,44,45,46,47,48,49,50,51,52,53,54,55] (Figure 1).

### 3.2. Basic Features Included in the Study

The basic characteristics of the included studies are summarized in Table 1. Thirty-two studies included data on total CSF tau protein and 24 on p-tau. Regarding study design, seven studies were prospective, three were retrospective, one was cross-sectional and the remaining studies did not have data available on the study design. Twenty-five of the studies used ELISA, three studies implemented INNO-BIA AlzBio3, and the remaining studies used other methods (Table 1).

### 3.3. Quality Evaluation of Included Studies

**Figure 2:**
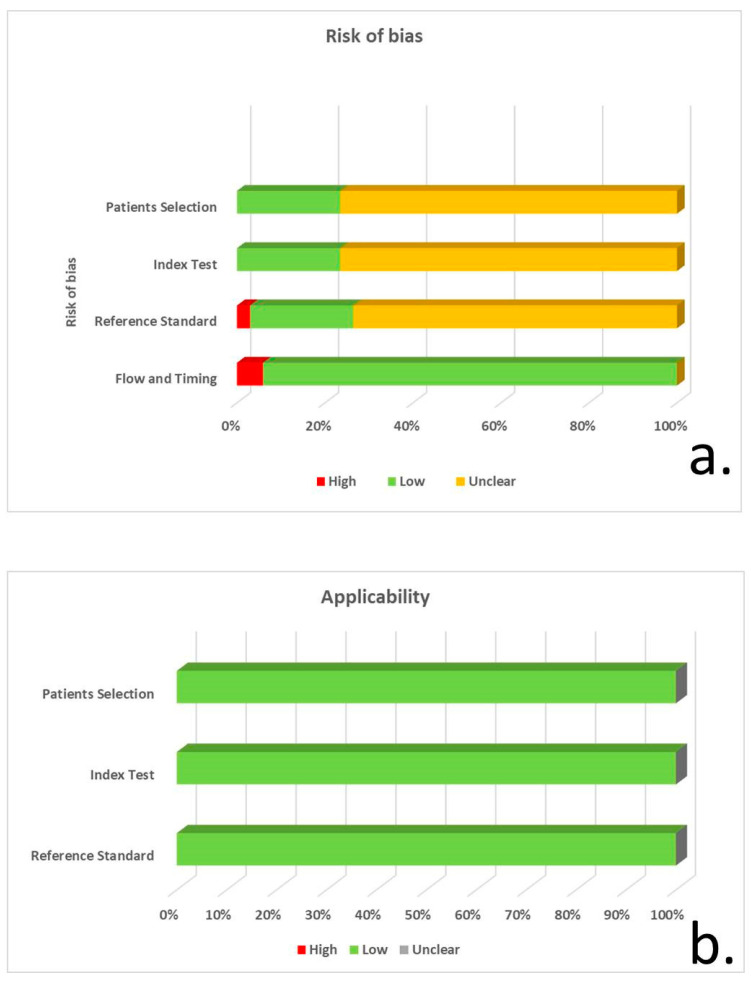
QUADAS-2 tool for risk of bias (**a**) and concerns on applicability of studies (**b**), for patient selection, index test, reference standard and flow/timing.

Based on the QUADAS-2 tool, 26 of the 34 studies did not explicitly specify the consecutive/random selection of patients, resulting in an unclear risk of bias regarding patient selection. Eight studies had a low risk of bias due to an unspecified consecutive/random sample selection. Only 12 studies reported blinding regarding CSF tau quantification. Despite this shortcoming, the risk of bias was considered low in all the studies, since tau measurements cannot be manipulated. The risk of bias for the reference standard and flow/timing was generally low (Figure 2, Supplementary File S3).

### 3.4. Results of Meta-Analysis

#### 3.4.1. Behavioral Variant Frontotemporal Dementia (bvFTD)

Eighteen studies included data on CSF t-tau in bvFTD cohorts. In total, 1494 subjects (592 bvFTD patients and 902 control subjects) were included in the above-mentioned studies. The bvFTD cohort samples ranged from 11 to 68 patients and the control cohorts ranged from 12 to 181 subjects. The mean CSF t-tau ranged from 80.1 to 1182 pg/mL in the bvFTD cohorts and from 20.0 to 622 pg/mL in the control subjects. The mean age in the bvFTD cohorts varied from 54.2 to 70.5 years, the mean disease duration varied from 2.3 to 5.3 years and the male-to-female ratio from 0.40 to 19. The mean age in the control cohorts varied from 56.5 to 70.1 years and the male-to-female ratio ranged from 0.20 to 2.5.

**Figure 3:**
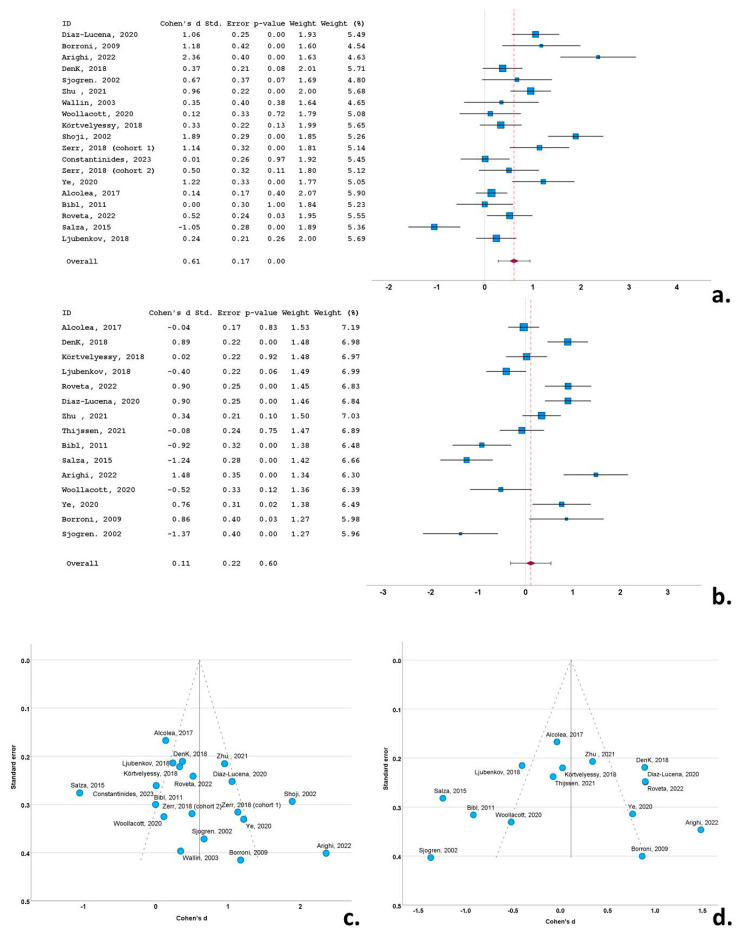
Forest plots of Cohen’s *d* of bvFTD studies for CSF total tau protein (**a**); CSF phosphorylated tau protein (**b**); funnel plots to illustrate possible publication bias for CSF total tau protein (**c**); CSF phosphorylated tau protein (**d**). For citations of studies refer to Table 1.

A total of 9 of the 19 cohorts (included in the 18 studies) with CSF t-tau data reported significantly higher levels of t-tau in the bvFTD cohorts compared to the control subjects. A single study reported significantly decreased t-tau in the bvFTD group compared to the control group. Ten studies did not report significant differences between the bvFTD and control groups. Cohen’s *d* ranged from −1.05 to 2.36. The overall Cohen’s *d* for t-tau was 0.61 (SE = 0.17; *p* < 0.001) (Figure 3a).

Fifteen studies included data on CSF p-tau in bvFTD cohorts. A total of 1040 subjects (474 bvFTD patients and 566 control subjects) were included in these studies. The bvFTD cohort samples ranged from 14 to 68 patients and the control cohorts ranged from 12 to 83 subjects. The mean CSF p-tau ranged from 9.1 to 100.7 pg/mL in the bvFTD cohorts and from 15.9 to 55 pg/mL in the control subjects. The mean age in the bvFTD cohorts varied from 54.2 to 70.5 years, the mean disease duration varied from 2.3 to 5.3 years and the male-to-female ratio ranged from 0.40 to 19. The mean age in the control cohorts varied from 56.5 to 70.1 years and the male-to-female ratio ranged from 0.21 to 2.5.

A total of 6 of the 15 cohorts with CSF p-tau data reported significantly higher levels of p-tau in the bvFTD cohorts compared to the control subjects. Three studies reported significantly decreased p-tau in the bvFTD group compared to the control group and six studies did not report significant differences between the two groups. Cohen’s *d* ranged from −1.37 to 1.48. Overall Cohen’s *d* for p-tau was 0.11 (SE = 0.22; *p* = 0.60) (Figure 3b).

Overall, Cohen’s *d* for p-tau was 0.11 (SE = 0.22; *p* = 0.60) (Figure 3b).

#### 3.4.2. Progressive Supranuclear Palsy (PSP)

Thirteen studies included data on CSF t-tau in PSP cohorts. A total of 1061 subjects (380 PSP patients and 681 control subjects) were included in these studies. The PSP cohort samples ranged from 14 to 50 patients and the control cohorts ranged from 14 to 181 subjects. The mean CSF t-tau ranged from 55 to 365.8 pg/mL in the PSP cohorts and from 20.0 to 365 pg/mL in the tcontrol subjects. The mean age in the PSP cohorts varied from 62 to 75 years, the mean disease duration varied from 2.1 to 8.0 years and the male-to-female ratio ranged from 0.75 to 1.92. The mean age in the control cohorts varied from 45.5–68.8 years and the male-to-female ratio ranged from 0.69 to 2.36.

**Figure 4:**
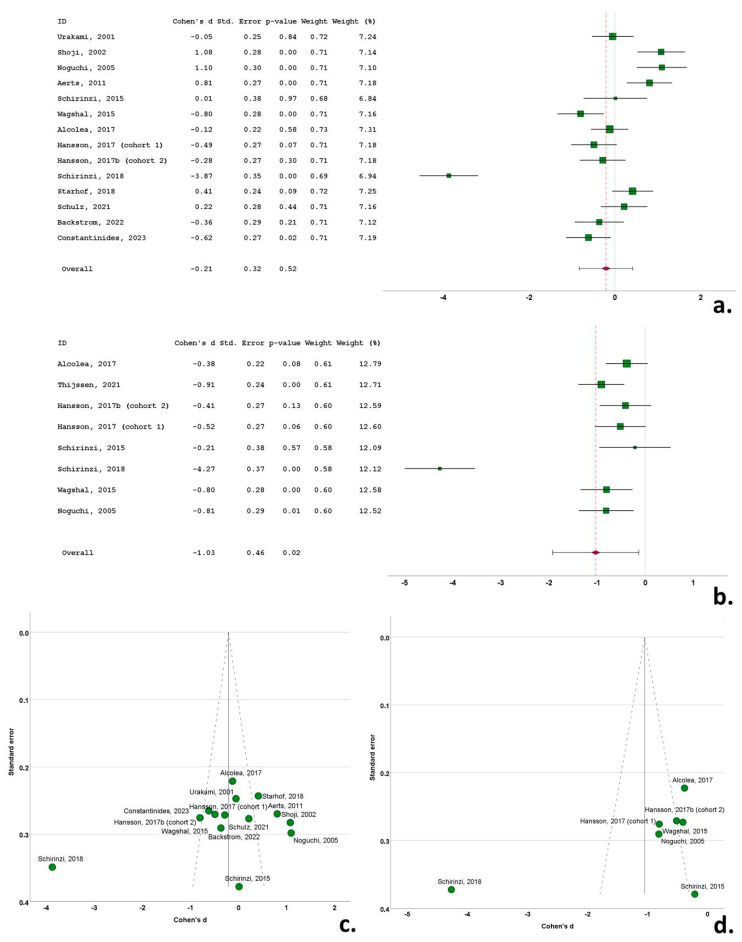
Forest plots of Cohen’s *d* of PSP studies for CSF total tau protein (**a**); CSF phosphorylated tau protein (**b**); funnel plots to illustrate possible publication bias for CSF total tau protein (**c**); CSF phosphorylated tau protein (**d**). For citations of studies refer to Table 1.

Three of the 14 cohorts (included in the 13 studies) with CSF t-tau data reported significantly higher levels of t-tau in the PSP cohorts compared to the control subjects. Three studies reported significantly decreased t-tau in the PSP group compared to the control group and eight studies did not report significant differences between the PSP and control groups. Cohen’s *d* ranged from −3.87 to 1.10. The overall Cohen’s *d* for t-tau was −0.21 (SE = 0.32; *p* = 0.52) (Figure 4a).

Seven studies included data on CSF p-tau in the PSP cohorts. A total of 555 subjects (200 PSP patients and 355 control subjects) were included in these studies. The PSP cohort samples ranged from 14 to 39 patients and the control cohorts ranged from 14 to 76 subjects. The mean CSF p-tau ranged from 14.5 to 43.1 pg/mL in the PSP cohorts and from 20.9 to 52.2 pg/mL in the control subjects. The mean age in the PSP cohorts varied from 67.9 to 72 years, the mean disease duration was from 2.3 to 6.3 years and the male-to-female ratio was from 0.75 to 1.29. The mean age in the control cohorts varied from 51.7 to 68.4 years and the male-to-female ratio ranged from 0.69 to 2.36.

Four of the eight cohorts (included in the seven studies) with CSF p-tau data reported significantly lower levels of p-tau in the PSP cohorts compared to the control subjects, with the remaining studies not reporting significant differences between the two groups. Cohen’s *d* ranged from −4.27 to −0.21. The overall Cohen’s *d* for p-tau was −1.03 (SE = 0.46; *p* = 0.02) (Figure 4b).

#### 3.4.3. Corticobasal Syndrome (CBS)

Nine studies included data on CSF t-tau in CBS cohorts. A total of 609 subjects (175 CBS patients and 434 control subjects) were included in these studies. The CBS cohort samples ranged from 11 to 45 patients and control cohorts from 12 to 181 subjects. The mean CSF t-tau ranged from 70.6 to 485.1 pg/mL in the CBS cohorts and from 20.0 to 365 pg/mL in the control subjects. The mean age in the CBS cohorts varied from 59.8 to 72.6 years, the mean disease duration ranged from 2.0 to 5.0 years and the male-to-female ratio ranged from 0.20 to 1.19. The mean age in the control cohorts varied from 57.0 to 70.5 years and the male-to-female ratio ranged from 0.69 to 2.33.

**Figure 5:**
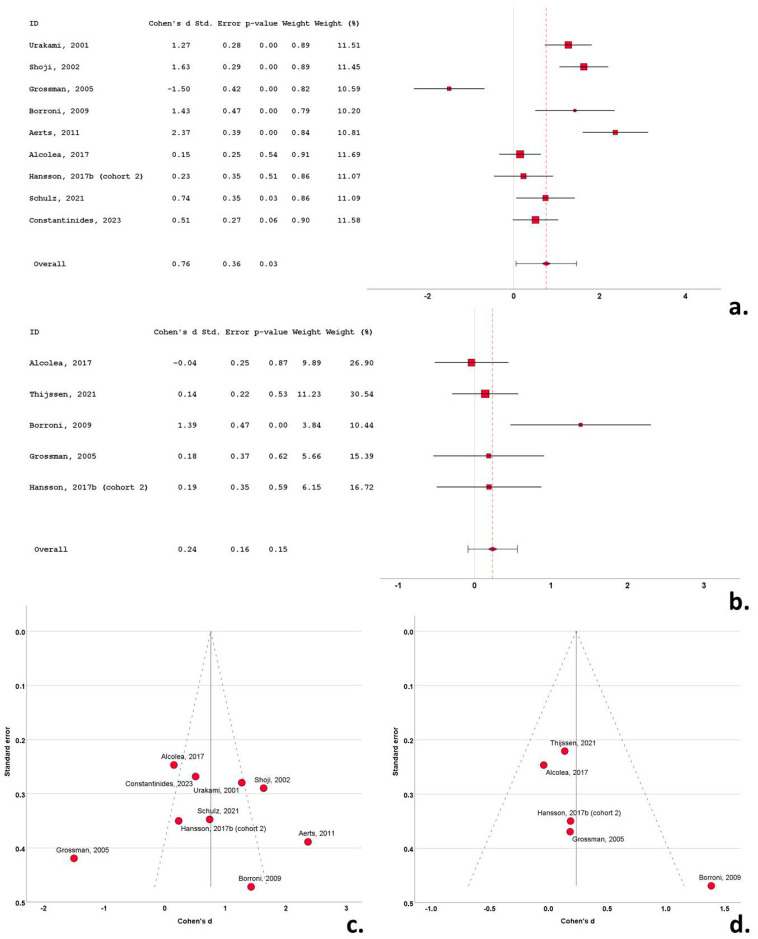
Forest plots of Cohen’s *d* of CBS studies for CSF total tau protein (**a**); CSF phosphorylated tau protein (**b**); funnel plots to illustrate possible publication bias for CSF total tau protein (**c**); CSF phosphorylated tau protein (**d**). For citations of studies refer to Table 1.

Five of the nine cohorts with CSF t-tau data reported significantly higher levels of t-tau in the CBS cohorts compared to the control subjects, with a single study reporting lower levels of t-tau in the CBS cohorts compared to controls and five studies reporting no between-group differences. Cohen’s *d* ranged from −1.50 to 2.37. The overall Cohen’s *d* for t-tau was 0.76 (SE = 0.36; *p* = 0.03) (Figure 5a).

Five studies included data on CSF p-tau in the CBS cohorts. A total of 272 subjects (97 CBS patients and 175 control subjects) were included in these studies. The CBS cohort samples ranged from 11 to 36 patients and the control cohorts ranged from 12 to 76 subjects. The mean CSF p-tau ranged from 22.5 to 91.3 pg/mL in the CBS cohorts and from 20.9 to 50.1 pg/mL in the control subjects. The mean age in the CBS cohorts varied from 59.8 to 72.6 years, the mean disease duration ranged from 3.7 to 4.6 years and the male-to-female ratio ranged from 0.20 to 1.19. The mean age in the control cohorts varied from 60.2 to 70.5 years and the male-to-female ratio ranged from 0.69 to 1.17.

Four of the five cohorts with CSF p-tau data did not report any difference in the p-tau CSF levels between the CBS patients and control subjects, with a single study reporting an increase in the p-tau CSF levels in the CBS compared to the control subjects. Cohen’s *d* ranged from −0.04 to −1.39. The overall Cohen’s *d* for p-tau was 0.24 (SE = 0.16; *p* = 0.15) (Figure 5b).

#### 3.4.4. Non-Fluent Agrammatic Primary Progressive Aphasia (nfa-PPA)

Six studies included data on CSF t-tau in nfa-PPA cohorts. A total of 390 subjects (112 nfa-PPA patients and 278 control subjects) were included in these studies. The nfa-PPA cohort samples ranged from 11 to 26 patients and the control cohorts ranged from 13 to 83 subjects. The mean CSF t-tau ranged from 80.1 to 490.6 pg/mL in the nfa-PPA cohorts and from 72 to 338.8 pg/mL in the control subjects. The mean age in the nfa-PPA cohorts varied from 67.7 to 72.8 years, the mean disease duration ranged from 2.3 to 4.2 years and the male-to-female ratio ranged from 0.55 to 1.36. The mean age in the control cohorts varied from 58.0 to 70.5 years and the male-to-female ratio ranged from 0.64 to 0.69.

**Figure 6:**
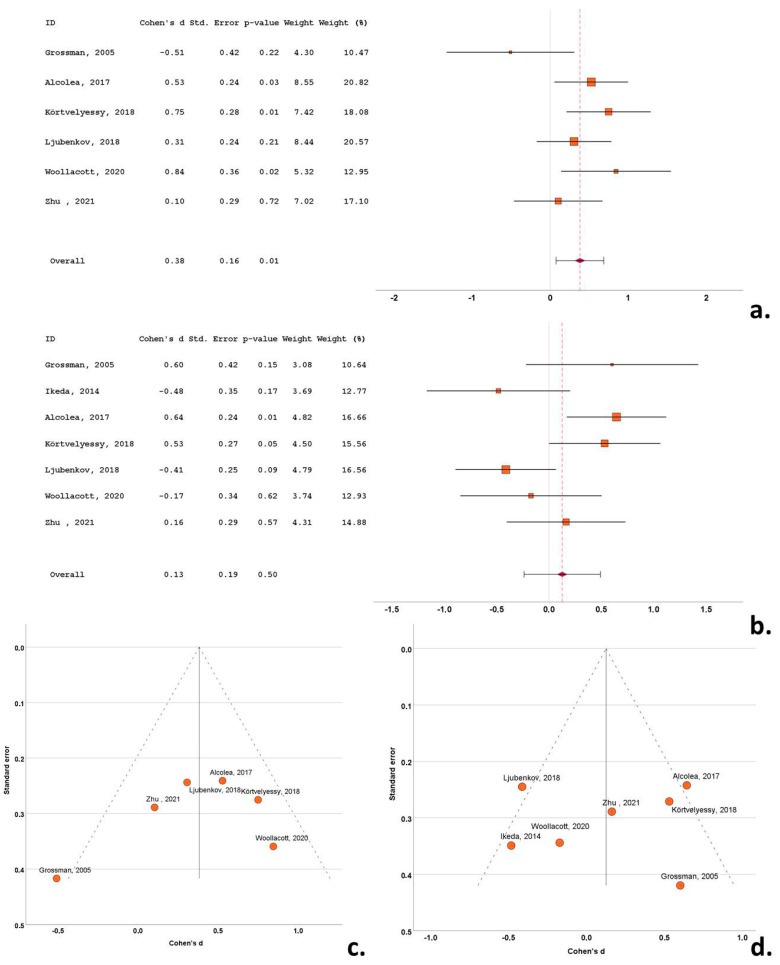
Forest plots of Cohen’s *d* of nfa-PPA studies for CSF total tau protein (**a**); CSF phosphorylated tau protein (**b**); funnel plots to illustrate possible publication bias for CSF total tau protein (**c**); CSF phosphorylated tau protein (**d**). For citations of studies refer to Table 1.

Three of the six cohorts with CSF t-tau data reported significantly higher levels of t-tau in the nfa-PPA cohorts compared to the control subjects, with the remaining three studies reporting no between-group differences. Cohen’s *d* ranged from −0.51 to 0.84. The overall Cohen’s *d* for t-tau was 0.38 (SE = 0.16; *p* = 0.01) (Figure 6a).

Seven studies included data on CSF p-tau in the nfa-PPA cohorts. A total of 450 subjects (122 nfa-PPA patients and 328 control subjects) were included in these studies. The nfa-PPA cohort samples ranged from 10 to 26 patients and the control cohorts ranged from 13 to 83 subjects. The mean CSF p-tau ranged from 21.7 to 64.6 pg/mL in the nfa-PPA cohorts and from 23.5 to 53.1 pg/mL in the control subjects. The mean age in the nfa-PPA cohorts varied from 66.8 to 72.8 years, the mean disease duration ranged from 2.3 to 5.6 years and the male-to-female ratio ranged from 0.55 to 1.36. The mean age in the control cohorts varied from 58.0 to 70.5 years and the male-to-female ratio ranged from 0.64 to 0.69.

A single study reported an increase in CSF p-tau in the nfa-PPA group compared to the control groups, with the remaining studies reporting no between-group differences. Cohen’s *d* ranged from −0.48 to −0.64. The overall Cohen’s *d* for p-tau was 0.13 (SE = 0.19; *p* = 0.50) (Figure 6b).

#### 3.4.5. Tauopathies

Four studies included data on CSF t-tau in the tauopathy cohorts. A total of 338 subjects (123 tauopathy patients and 215 control subjects) were included in these studies. The tauopathy cohort samples ranged from 13 to 50 patients and the control cohorts ranged from 13 to 65 subjects. The mean CSF t-tau ranged from 43 to 290 pg/mL in the tauopathy cohorts and from 46 to 249 pg/mL in the control subjects. The mean age in the tauopathy cohorts varied from 60.7 to 65.0 years and the male-to-female ratio ranged from 1.08 to 1.86. The mean age in the control cohorts varied from 59.0 to 68.1 years and the male-to-female ratio ranged from 0.51 to 1.19.

**Figure 7:**
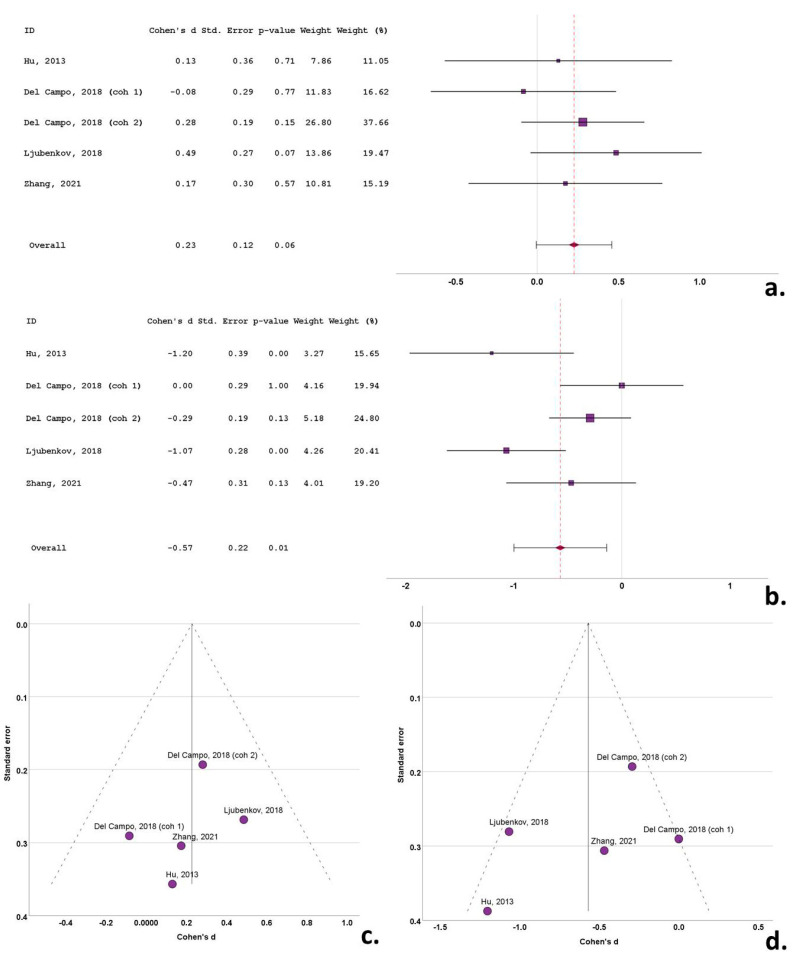
Forest plots of Cohen’s *d* of tauopathy studies for CSF total tau protein (**a**); CSF phosphorylated tau protein (**b**); funnel plots to illustrate possible publication bias for CSF total tau protein (**c**); CSF phosphorylated tau protein (**d**). For citations of studies refer to Table 1.

None of the five cohorts (included in the four studies) with CSF t-tau data reported any significant between-group differences. Cohen’s *d* ranged from −0.08 to 0.49. The overall Cohen’s *d* for t-tau was 0.23 (SE = 0.12; *p* = 0.06) (Figure 7a).

The studies reporting data on CSF p-tau in the tauopathy cohorts were identical to the studies including data on t-tau in the tauopathies. Three studies reported a decrease in the CSF p-tau in the tauopathy group compared to the control groups, with the remaining studies reporting no between-group differences. Cohen’s *d* ranged from −1.20 to 0.00. The overall Cohen’s *d* for p-tau was −0.57 (SE = 0.22; *p* = 0.01) (Figure 7b).

### 3.5. Heterogeneity

The presence/absence of heterogeneity was assessed qualitatively by the *Q* statistic. The *I^2^* statistic was implemented to quantify the between-study heterogeneity.

In the bvFTD studies, both CSF t-tau (*Q* statistic *p*-value < 0.001; *I^2^* = 0.86) and p-tau (*Q* statistic *p*-value < 0.001; *I^2^* = 0.91) exhibited high heterogeneity. The same pattern was evident in the PSP cohorts for CSF t-tau and p-tau (*Q* statistic *p*-value < 0.001; *I^2^* = 0.95 for both). In the CBS cohorts, the heterogeneity was high for CSF t-tau (*Q* statistic *p*-value < 0.001; *I^2^* = 0.91) but was absent qualitatively for p-tau (*Q* statistic *p*-value = 0.11; *I^2^* = 0.30). For the nfa-PPA cohorts, CSF t-tau exhibited no qualitative heterogeneity among the studies (Q statistic *p*-value = 0.10), whereas p-tau exhibited high heterogeneity (*Q* statistic *p*-value = 0.01; *I^2^* = 0.63). The same pattern was evident for the tauopathy studies, with homogeneity among the t-tau studies (*Q* statistic *p*-value = 0.69; *I^2^* = 0.00) but high heterogeneity among the p-tau studies (*Q* statistic *p*-value = 0.02; *I^2^* = 0.67).

### 3.6. Publication Bias

Funnel plots exhibited a degree of asymmetry in the bvFTD, PSP and CBS groups for both t-tau and p-tau, indicative of publication bias. More specifically, in bvFTD studies publication bias was evident, with multiple outlying (out of the 95% confidence interval) studies of small size for t-tau [25,44] and for p-tau [53], possibly indicative of small study bias (Figure 3c,d). The same pattern was evident in the PSP studies, with a single study exhibiting a disproportionately large effect size for both t-tau and p-tau, despite the moderate size of study groups [35] (Figure 4c,d). This was also evident for small sized studies in CBS for t-tau [51] and for p-tau) [49] (Figure 5c,d). In contrast to this pattern, nfa-PPA and tauopathy studies had relatively symmetrical funnel plots (Figure 6c,d and Figure 7c,d).

The results of the Egger’ regression-based test, however, were not supportive of publication bias for bvFTD (t-tau:β0 = −0.54; −1.98 to 0.90; *p*-value = 0.44; p-tau:β0 = 0.48; −1.51 to 2.47; *p*-value = 0.61), PSP (t-tau:β0 = 2.94; −1.98 to 7.85; *p*-value = 0.22; p-tau:β0 = 2.44.; −3.36 to 8.25; *p*-value = 0.34), CBS (t-tau:β0 = 0.90; −3.52 to 5.31; *p*-value = 0.65; p-tau:β0 = −0.79.; −2.39 to 0.81; *p*-value = 0.21), nfa-PPA (t-tau:β0 = 1.22; −1.12 to 3.56; *p*-value = 0.22; p-tau:β0 = 0.24.; −2.50 to 2.98; *p*-value = 0.83) or tauopathy (t-tau:β0 = 0.55; −1.23 to 2.32; *p*-value = 0.40; p-tau:β0 = 0.54; −2.695 to 3.77; *p*-value = 0.63). The discrepancy between funnel plots and Egger’s regression test could be attributed to small-study effect on funnel plots, which is common in studies of rare diseases.

## 4. Discussion

Over the past decade, there has been a pivotal shift in the conceptual framework of cognitive and movement disorders, from clinical entities to biologically defined entities. This shift was driven by advances in biomarkers in the field of AD, which allowed for the early and accurate in vivo diagnosis of amyloidosis and tau pathology, even in the preclinical stages of AD [11]. Recently, a similar approach has been adopted for Parkinson’s disease, with the development of a-synuclein seeding amplification assays [56,57].

The availability of biomarkers for AD has significantly advanced our knowledge on the pathophysiological mechanisms underlying AD. On a clinical level, AD biomarkers have assisted in recognizing the atypical manifestations of AD, as illustrated in established diagnostic criteria [58]. Importantly, clinical studies based on biomarkers have allowed for the development of possibly disease-modifying agents for AD [59].

Despite these advances, biomarkers for other common neurodegenerative disorders, including tauopathies and TDP-proteinopathies are lacking. For FTLD in particular, where clinical and pathological heterogeneity is great, biomarkers of tau pathology or TDP pathology would be pivotal for advancing the field by facilitating the distinction of the two major pathologies in FTD.

In this review, we examined the diagnostic performance of CSF t-tau and p-tau in the four most common phenotypes of FTLD-tau: bvFTD, RS, CBS and nfa-PPA. Both t-tau and p-tau are established biomarkers in the field of AD of neurodegeneration and tau pathology, respectively [11]. Due to their availability, these markers have been extensively assessed in the context of diverse neurodegenerative disorders, including FTD.

The vast majority of relative studies in this meta-analysis included clinically diagnosed patients (i.e., based on clinical phenotype) without neuropathological confirmation. This is expected to result in cohorts with mixed pathologies. This presents a major obstacle to interpreting the results of this meta-analysis. Importantly, the possibility of including a tauopathy in a cohort depends on the clinical phenotype, with RS patients expected to almost exclusively have a 4R-tauopathy [10]. CBS and nfa-PPA patients are expected to harbor a tauopathy in the majority of cases, whereas tauopathies represent ~50% of bvFTD patients [7,8,9]. Additionally, we included data from patients with a definite tauopathy, based on pathology or the presence of a known pathogenic MAPT mutation.

An initial finding of this meta-analysis is that CSF p-tau was decreased in the PSP and tauopathy patients compared to the controls. This decrease was not present in the other phenotypes (bvFTD, CBS, nfa-PPA). This finding is of particular interest when taking into consideration that the PSP cohort is the most likely to almost exclusively include patients with tauopathy. The effect size, as measured by Cohen’s *d,* was large for PSP (~−1) and medium (~−0.6) for the tauopathy cohorts. However, for the PSP group in particular, this effect size could be partially driven by a single small-sized study with a disproportionally high effect size. Despite this decrease in CSF p-tau at the group level, the significant overlap between the patient groups and control groups precludes the use of CSF p-tau as a biomarker.

Interestingly, a similar pattern is observed in AD, where CSF amyloid beta with 42 amino acids (Ab42) is decreased in patients with amyloidosis. This has been attributed to the retention of Ab42 in the amyloid plaques, which results in the decreased availability of Ab42 in the CSF. Whether a similar mechanism mediates the relative decrease in CSF p-tau in tauopathies has not been validated to date. The admixture of AD patients (who are characterized by an increase in CSF p-tau), primarily in the bvFTD cohorts and secondarily in the CBS and nfa-PPA cohorts, could hypothetically cover a possible inherent decrease in p-tau in these disorders.

A second important finding of this meta-analysis was the increase in CSF t-tau in bvFTD, CBS and nfa-PPA. The effect size, as measured by Cohen’s *d*, was medium for bvFTD (~0.6) and nfa-PPA (~0.4) and large for CBS (~0.8). Interestingly, the PSP group and the tauopathy group did not exhibit such an increase. CSF t-tau is a non-specific marker of neuronal death/axonal injury, and is therefore increased in diverse conditions characterized by neuronal injury, such as AD, CJF, head trauma, etc. Thus, an initial hypothesis for the documented increase in t-tau in bvFTD, CBS and nfa-PPA could be that it reflects the ongoing neurodegeneration in these conditions. However, the absence of such an increase in PSP, a disorder with comparatively more rapid progression and poorer outcomes, is not consistent with this hypothesis. An alternative hypothesis is that this increase in CSF t-tau is driven by the admixture of AD patients in the bvFTD, CBS and nfa-PPA cohorts.

The findings of the present meta-analysis should be reviewed with caution. As illustrated in the heterogeneity analyses, there was significant heterogeneity among the studies in most cases. This heterogeneity is expected in meta-analyses of multiple, mostly small-sized studies, as is the case in most rare diseases. This heterogeneity is further evidenced by the great variability in cohort sizes, study designs, type of control groups used (i.e., neurological vs. healthy), and patient/control group demographics (age, disease duration, male/female ratio). An additional source of heterogeneity is the variability in pre-analytical and analytical factors of CSF biomarker measurements, as illustrated by the great range in CSF t-tau and p-tau levels in the control groups among the studies [60]. Analytical differences, including possible differences in capture and detecting antibodies, particularly for p-tau, may be the main cause for this heterogeneity. Likewise, the funnel plots indicated the presence of publication bias.

The present meta-analysis aimed to investigate the role of CSF t-tau and p-tau in the most common clinical manifestations of FTLD-tau. Although these markers cannot be used as biomarkers for the diagnosis of tauopathy, this study provides some support to the presence of an inherent decrease in p-tau in tauopathies. A single meta-analysis was previously performed on the subject [15]. This meta-analysis focused on a comparison between FTLD and Alzheimer’s disease regarding t-tau and p-tau. Additionally, in the FTLD group, patients with phenotypes not commonly related to tauopathy, such as semantic dementia, were included. This meta-analysis concluded that there may be an intermediate increase in t-tau in FTLD. However, due to differences in the study design, our findings cannot be directly compared to the findings of the previous meta-analysis. Given the shortcomings of a meta-analysis based on mostly small-sized, heterogeneous studies of clinically defined patients, these findings should be validated in larger cohorts of pathologically or genetically defined cohorts of patients with tauopathy. Importantly, novel approaches are needed in an effort to develop a clinically applicable biomarker for tauopathies. To this end, PET tau tracers and tau seeding amplification assays have provided some encouraging preliminary results [61,62,63].

## 5. Conclusions

The present meta-analysis indicates that primary tauopathies may exhibit an inherent decrease in CSF p-tau. Due to significant between-group overlap, CSF t-tau and p-tau cannot be implemented as biomarkers of tauopathy. Novel biomarkers with molecular specificity for tau are needed to facilitate the early and accurate in vivo diagnosis of tauopathies.

## Figures and Tables

**Table 1:** Characteristics of included studies. Study design: P: prospective; R: retrospective; C: cross-sectional; na: not available; FTD: bvFTD; PNFA: nfa-PPA; ctrl: control group.

Study N	Study	Study Design	Period of Recruitment	Study Origin	Method	Disease Groups	Tau Proteins	Main Findings
1	Urakami, 2001 [55]	na	na	Japan multicenter	ELISA	PSP; CBS	t-tau	t-tau: CBD > ctrl (*p* < 0.001); PSP ≈ ctrl
2	Shoji, 2002 [54]	na	na	Japan	ELISA	PSP; CBS; FTD	t-tau	t-tau: FTD > ctrl (*p* < 0.01); CBD > ctrl (*p* < 0.05) PSP ≈ ctrl
3	Sjogren, 2002 [53]	P	na	na	ELISA	FTD	t-tau; p-tau	p-tau: FTD < ctrl (*p* < 0.05)
4	Wallin, 2003 [52]	P	na	Sweden	ELISA	FTD	t-tau	t-tau: FTD ≈ ctrl
5	Grossman, 2005 [51]	P	na	USA	ELISA	CBS; PNFA	t-tau; p-tau	t-tau: CBS < ctrl (*p* < 0.01); PNFA ≈ ctrl p-tau: CBS ≈ ctrl; PNFA ≈ ctrl
6	Noguchi, 2005 [50]	na	na	na	ELISA (Innotest)	PSP	t-tau; p-tau	t-tau PSP ≈ ctrl, p-tau PSP ≈ ctrl,
7	Borroni, 2009 [49]	na	na	na	ELISA (Innotest)	FTD; CBS	t-tau; p-tau	t-tau: FTD > ctrl (*p* < 0.001); CBDS > ctrl (*p* < 0.05) p-tau: FTD > ctrl (*p* < 0.05); CBS ≈ ctrl
8	Aerts, 2011 [48]	R	1998–2007	Netherlands	ELISA (Innotest)	PSP; CBS	t-tau	t-tau: CBS > ctrl (*p* < 0.001); PSP > ctrl (*p* < 0.05) p-tau: CBS > ctrl (*p* < 0.001); PSP ≈ ctrl
9	Bibl, 2011 [47]	na	1999–2004	Germany	ELISA (Innotest)	FTD	t-tau;p-tau	t-tau: FTD ≈ ctrl p-tau: FTD < ctrl (*p* < 0.005)
10	Hu, 2013 [46]	P	na	USA	AlzBio3 Innogenetics	tauopathy	t-tau;p-tau	t-tau: FTLD ≈ ctrl p-tau: FTLD ≈ ctrl
11	Ikeda, 2014 [45]	na	na	Japan	TR-FRET immunoassay	PNFA	p-tau	p-tau: PNFA ≈ ctrl
12	Salza, 2015 [44]	na		France	ELISA (Innotest)	bvFTD	t-tau; p-tau	t-tau and p-tau: bvFTLD vs. ctrl: na
13	Schirinzi, 2015 [43]	na	2012–2014	Italy	ELISA	PSP	t-tau; p-tau	t-tau: PSP ≈ ctrl p-tau PSP ≈ ctrl
14	Wagshal, 2015 [42]	na	na	USA	INNO BIA AlzBio3	PSP	t-tau; p-tau	Total tau and p-tau PSP < ctrl (*p* < 0.05)
15	Alcolea, 2017 [41]	na	na	Multicenter	ELISA (Innotest)	bvFTD; CBS; PSP; PNFA	t-tau; p-tau	t-tau: PSP, CBS, bvFTD, PNFA ≈ ctrl p-tau: PSP, CBS, bvFTD, PNFA ≈ ctrl
16	Hansson, 2017 (cohort 1) [40]	P	na	Biofinder study	ELISA	PSP	t-tau; p-tau	t-tau: PSP < ctrl (*p* < 0.05) p-tau: PSP < ctrl (*p* < 0.01)
16	Hansson 2007 (cohort 2) [40]	P	na	Biofinder study	ELISA	PSP; CBS	t-tau; p-tau	t-tau: PSP ≈ ctrl; CBS ≈ ctrl p-tau: PSP < ctrl (*p* < 0.01); CBS ≈ ctrl p-tau
17	Del Campo, 2018 (cohort 1) [39]	na	na	Multicenter	INNO-BIA AlzBio3, ELISA (Innotest)	tauopathy	t-tau; p-tau	t-tau: FTLD-tau ≈ ctrl p-tau: FTLD-tau ≈ ctrl
17	Del Campo, 2018 (cohort 2) [39]	na	na	Multicenter	na	tauopathy	t-tau; p-tau	t-tau: FTLD-tau ≈ ctrl p-tau: FTLD-tau ≈ ctrl
18	DenK, 2018 [38]	na	na	German	ELISA	FTD	t-tau; p-tau	t-tau: FTD ≈ ctrl p-tau: FTD > ctrl (*p* < 0.05)
19	Körtvelyessy, 2018 [37]	na	na	Germany	ELISA	FTD; PNFA	t-tau; p-tau	t-tau: ≈ ctrl t-tau: PNFA > ctrl (*p* < 0.05)
20	Ljubenkov, 2018 [36]	na	2009–2015	Multicenter	INNO-BIA AlzBio3	FTD; PNFA; tauopathy	t-tau; p-tau	t-tau: FTD ≈ ctrl; PNFA ≈ ctrl p-tau: FTD ≈ ctrl; PNFA ≈ ctrl
21	Schirinzi, 2018 [35]	P	2014–2017	Italy	ELISA	PSP	t-tau; p-tau	t-tau: PSP < ctrl (*p* = 0.03) p-tau: PSP < ctrl (*p* = 0.02)
22	Starhof, 2018 [34]	na	2007–2015	Denmark	ELISA (Innotest)	PSP	t-tau	t-tau: PSP ≈ ctrl
23	Zerr, 2018 (cohort 1) [33]	na	na	Portugal	ELISA (Innotest)	FTD	t-tau	t-tau: FTD ≈ ctrl
23	Zerr, 2018 (cohort 2) [33]	na	na	Portugal	ELISA (Innotest)	FTD	t-tau	t-tau: FTD ≈ ctrl
24	Diaz-Lucena, 2020	na	na	Germany, Portugal	ELISAs (INNOTEST)	FTD	t-tau; p-tau	t-tau: FTD > ctrl (*p* < 0.001) p-tau FTD > ctrl (*p* < 0.01)
25	Woollacott, 2020 [31]	na	na	UK	ELISA (Innotest)	FTD; PNFA	t-tau; p-tau	t-tau: FTLD, PNFA vs. ctrl: na p-tau: FTLD, PNFA vs. ctrl: na
26	Ye, 2020 [30]	na	2015–2019	China	ELISA	FTD	t-tau; p-tau	t-tau: FTD > ctrl (*p* < 0.001) p-tau: FTD > ctrl (*p* < 0.05)
27	Schulz, 2021 [29]	C	na	Germany	Human Neurology 4-Plex	PSP; CBS	t-tau	t-tau: PSP > ctrl (*p* = 0.047) t-tau: CBS > ctrl (*p* = 0.023)
28	Thijssen, 2021 [28]	R	na	USA, Canada	Elecsys	FTD; PSP; CBS	p-tau	p-tau: FTD, PSP, CBS ≈ ctrl
29	Zhang, 2021 [27]	na	1997- 2014	USA	INNO-BIA AlzBio3	tauopathy (=autopsy or genetic)	t-tau; p-tau	t-tau: tauopathy ≈ ctrl p-tau: tauopathy ≈ ctrl
30	Zhu, 2021 [26]	na	na	SPIN cohort	Lumipulse	FTD; PNFA	t-tau; p-tau	t-tau: FTD, PNFA ≈ ctrl p-tau: FTD, PNFA ≈ ctrl
31	Arighi, 2022 [25]	na	2012–2020	Italy	ChemiLuminescence Enzyme ImmunoAssay (CLEIA)	FTD	t-tau; p-tau	t-tau and p-tau: bvFTLD vs. ctrl: na
32	Backstrom, 2022 [24]	P	2004–2009	Sweden	ELISA (Innotest)	PSP	t-tau	t-tau: PSP ≈ ctrl
33	Roveta, 2022 [23]	na	na	Italy	ELISA (Innotest)	FTD	t-tau; p-tau	t-tau: FTD ≈ ctrl p-tau FTD > ctrl (*p* = 0.001)
34	Constantinides, 2023 [22]	R	2011–2021	Greece	ELISA (EUROIMMUN)	FTD; PSP; CBS	t-tau	t-tau and p-tau: bvFTLD, PSP, CBS vs. ctrl: na

## Data Availability

The raw data supporting the conclusions of this article will be made available by the authors on request.

## Supplementary Materials

The following supporting information can be downloaded at https://www.mdpi.com/article/10.3390/biomedicines12081781/s1, Supplementary File S1: PRISMA checklist. Supplementary File S2: Table summarizing search strategy. Supplementary File S3: Quality assessment of studies, based on the QUADAS-2 test.
