# Supplementary material for: Cerebrospinal Fluid Total and Phosphorylated Tau Protein in Behavioral Variant Frontotemporal Dementia, Progressive Supranuclear Palsy, Corticobasal Syndrome and Non-Fluent Agrammatic Primary Progressive Aphasia: A Systematic Review and Meta-Analysis
Source: Biomedicines. 2024 Aug 6;12(8):1781. doi: 10.3390/biomedicines12081781 (PMC11351341; doi:10.3390/biomedicines12081781)
Supplement: Supplementary file 1 [file biomedicines-12-01781-s001.zip › Supplementary File S2.pdf]

A	B	C
Corticobasal	Biomarker*	Cerebrospinal fluid
Aphasia	P-tau	Fluid
Supranuclear	Ptau	CSF
Richardson	Tau	
Behavioral	Phosphorylated tau	
Behavioural		
Frontotemporal		
Agrammatic		
Tauopath*		

Supplementary File S2. Terms used in literature searches. Each column represents an OR clause. Columns A, B and C were combined in an AND clause. The searches were limited to title or abstract.
