# Supplementary material for: Cerebrospinal Fluid Total and Phosphorylated Tau Protein in Behavioral Variant Frontotemporal Dementia, Progressive Supranuclear Palsy, Corticobasal Syndrome and Non-Fluent Agrammatic Primary Progressive Aphasia: A Systematic Review and Meta-Analysis
Source: Biomedicines. 2024 Aug 6;12(8):1781. doi: 10.3390/biomedicines12081781 (PMC11351341; doi:10.3390/biomedicines12081781)
Supplement: Supplementary file 1 [file biomedicines-12-01781-s001.zip › Supplementary File S3.pdf]

	Patient Selection				Index Test			Reference Standard					Flow and Timing			
	Consecutive / random sample	Innapropriate exclusions?	ROB	App	Test assessed blindly	ROB	App	Is the RS likely to correctly classify participants	At least 2 judges of ref standard?	Ref. standard assessed without knowledge of index test?	ROB	App	All patients received RS	All patients received same RS	All patient included?	ROB
Aerts, 2011	?	☺	?	☺	?	?	☺	☺	☹	?	☹	☺	☺	☺	☺	☺
Alcolea, 2017	?	☺	?	☺	?	?	☺	☺	?	?	?	☺	☺	☺	☺	☺
Arighi, 2022	?	☺	?	☺	?	?	☺	☺	?	?	?	☺	☺	☺	☺	☺
Backstrom, 2022	☺	☺	☺	☺	?	?	☺	☺	☺	?	?	☺	☺	☺	☺	☺
Bibl, 2011	?	☺	?	☺	☺	☺	☺	☺	☺	☺	☺	☺	☺	☺	☹	☹
Borroni, 2009	☺	☺	☺	☺	☺	☺	☺	☺	?	?	?	☺	☺	☺	☺	☺
Constantinides, 2023	?	☺	?	☺	?	?	☺	☺	?	?	?	☺	☺	☺	☺	☺
Del Campo, 2018	?	☺	?	☺	?	?	☺	☺	?	?	?	☺	☺	☹	☺	☹
DenK, 2018	?	☺	?	☺	☺	☺	☺	☺	?	?	?	☺	☺	☺	☺	☺
Diaz-Lucena, 2020	?	☺	?	☺	☺	☺	☺	☺	?	?	?	☺	☺	☺	☺	☺
Grossman, 2005	?	☺	?	☺	?	?	☺	☺	?	☺	?	☺	☺	☺	☺	☺
Hansson, 2017	☺	☺	☺	☺	?	?	☺	☺	?	☺	?	☺	☺	☺	☺	☺
Hu, 2013	☺	☺	☺	☺	☺	☺	☺	☺	☺	☺	☺	☺	☺	☺	☺	☺
Ikeda, 2014	?	☺	?	☺	?	?	☺	☺	?	☺	☺	☺	☺	☺	☺	☺
Körtvelyessy, 2018	?	☺	?	☺	☺	?	☺	☺	?	?	?	☺	☺	☺	☺	☺
Ljubenkov, 2018	?	☺	?	☺	☺	?	☺	☺	?	?	?	☺	☺	☺	☺	☺
Noguchi, 2005	?	☺	?	☺	☺	?	☺	☺	?	?	?	☺	☺	☺	☺	☺
Roveta, 2022	☺	☺	☺	☺	?	☺	☺	☺	?	?	?	☺	☺	☺	☺	☺
Salza, 2015	?	☺	?	☺	☺	?	☺	☺	☺	☺	☺	☺	☺	☺	☺	☺
Schirinzi, 2015	?	☺	?	☺	?	?	☺	☺	?	☺	?	☺	☺	☺	☺	☺

**Schirinzi, 2018**	?	☺	?	☺	?	?	☺	☺	?	☺	?	☺	☺	☺	☺	☺
**Schulz, 2021**	?	☺	?	☺	?	?	☺	☺	?	☺	?	☺	☺	☺	☺	☺
**Shoji, 2002**	?	☺	?	☺	?	?	☺	☺	?	☺	?	☺	☺	☺	☺	☺
**Sjogren. 2002**	☺	☺	☺	☺	☺	☺	☺	☺	☺	☺	☺	☺	☺	☺	☺	☺
**Starhof, 2018**	?	☺	?	☺	?	?	☺	☺	?	☺	☺	☺	☺	☺	☺	☺
**Thijssen, 2021**	?	☺	?	☺	☺	?	☺	☺	?	?	?	☺	☺	?	☺	☺
**Urakami, 2001**	?	☺	?	☺	?	?	☺	☺	?	?	?	☺	☺	☺	☺	☺
**Wagshal, 2015**	?	☺	?	☺	?	?	☺	☺	?	?	?	☺	☺	☺	☺	☺
**Wallin, 2003**	☺	☺	☺	☺	?	?	☺	☺	?	?	?	☺	☺	☺	☺	☺
**Woollacott, 2020**	☺	☺	☺	☺	?	?	☺	☺	☺	?	?	☺	☺	☺	?	☺
**Ye, 2020**	?	☺	?	☺	?	?	☺	☺	?	☺	?	☺	☺	☺	☺	☺
**Zerr, 2018**	?	☺	?	☺	☺	☺	☺	☺	☺	☺	☺	☺	☺	☺	?	☺
**Zhang, 2021**	?	☺	?	☺	?	?	☺	☺	☺	☺	☺	☺	☺	☺	?	☺
**Zhu , 2021**	?	☺	?	☺	?	?	☺	☺	?	☺	?	☺	☺	☺	☺	☺

Supplementary File S3. Quality assessment of studies, based on the QUADAS-2 test; ROB: Risk of Bias; App: Concerns regarding applicability; ☺: low; ☹: high; ?: unclear
